# Targeting the PD-1/PD-L1 Pathway in Renal Cell Carcinoma

**DOI:** 10.3390/ijms20071692

**Published:** 2019-04-04

**Authors:** Solène-Florence Kammerer-Jacquet, Antoine Deleuze, Judikaël Saout, Romain Mathieu, Brigitte Laguerre, Gregory Verhoest, Frédéric Dugay, Marc-Antoine Belaud-Rotureau, Karim Bensalah, Nathalie Rioux-Leclercq

**Affiliations:** 1 Department of Pathology, University Hospital, 35000 Rennes, France; nathalie.rioux-leclercq@chu-rennes.fr; 2 Université Rennes, Inserm, EHESP (Ecole des Hautes Etudes en Santé Publique), Irset (Institut de recherche en santé, environnement et travail) - UMR_S 1085, F-35000 Rennes, France; judikael.saout@univ-rennes1.fr (J.S.); romain.mathieu@chu-rennes.fr (R.M.); frederic.dugay@chu-rennes.fr (F.D.); marc-antoine.belaud-rotureau@chu-rennes.fr (M.-A.B.-R.); 3 Department of Medical Oncology, Centre Eugene Marquis, 35000 Rennes, France; acjdeleuze@gmail.com (A.D.); b.laguerre@rennes.unicancer.fr (B.L.); 4 Department of Urology, University Hospital, 35000 Rennes, France; gregory.verhoest@chu-rennes.fr (G.V.); karim.bensalah@chu-rennes.fr (K.B.); 5 Department of Cytogenetics, University Hospital, F-35000 Rennes, France

**Keywords:** immunotherapy, immune checkpoint inhibition, renal cell carcinoma, PD-1, PD-L1, biomarker, tumour mutational burden

## Abstract

Renal cell carcinoma encompass distinct diseases with different pathologic features and distinct molecular pathways. Immune checkpoint inhibitors targeting the programmed death receptor ligand 1 (PD-L1)/programmed death receptor 1 (PD-1) pathway alone or in combination have greatly changed clinical management of metastatic renal cell carcinoma, now competing with antiangiogenic drugs in monotherapy for first-line treatment. However, long-term response rates are low, and biomarkers are needed to predict treatment response. Quantification of PD-L1 expression by immunohistochemistry was developed as a promising biomarker in clinical trials, but with many limitations (different antibodies, tumour heterogeneity, specimens, and different thresholds of positivity). Other biomarkers, including tumour mutational burden and molecular signatures, are also developed and discussed in this review.

## 1. Introduction

Renal cell carcinoma (RCC) represents 2% to 3% of all cancers, corresponding to 338,000 new cases diagnosed each year worldwide [1]. Around 30% of patients will present metastatic disease at the time of diagnosis and metastases are found in 30% to 40% of those initially treated in curative intent. If the incidence and mortality tend to decrease in Western Europe [2], recent data from the United States show a continued increase in incidence [3].

Clear cell renal cell carcinomas account for about 75% of RCC [1]. The other histologies are numerous according to the last WHO classification and mainly include the following entities: Papillary (20%) and chromophobe RCC (5%), which are the most frequent non-clear cell RCC (nccRCC), collecting duct carcinoma; medullar RCC, and translocation-associated RCC, with distinct pathologic features and different molecular alterations [4]. Among them, collecting duct carcinoma has been identified as an immunogenic tumour characterized by the upregulation of genes involved in the immune response [5].

The management and treatment of metastatic renal cell cancer have undergone major improvements over the last 20 years. The development of targeted therapies, including vascular endothelial growth factor (VEGF) pathway inhibitors, mammalian target of rapamycin (mTOR) inhibitors, and, more recently, the emergence of immune checkpoint inhibitors (ICI), such as anti-programmed death receptor 1 (PD-1), anti-programmed death receptor ligand 1 (PD-L1), and anti-cytotoxic T lymphocytes antigen 4 (CTLA-4), have led to a wide treatment landscape remodelling, targeting the tumour micro-environment (Table 1).

The advent of targeted therapies highlights the central role of the tumour micro-environment. The role of angiogenesis was already well known in ccRCC, with the VHL inactivation pathway leading to angiogenesis. The immune response with ICI is a more recent strategy. There is also a biological rationale to use them in association.

In this comprehensive review, we will discuss: (1) the histologic subtypes and the molecular pathways related, (2) the anti-tumour immune response and its interaction with angiogenesis, (3) the current landscape of targeted immunotherapy in RCC, and (4) the predictive biomarkers in RCC, including their limitations and the perspectives.

## 2. Histologic Subtypes and Related Molecular Pathways

### 2.1. Clear Cell Renal Cell Carcinoma

**Figure 1:**
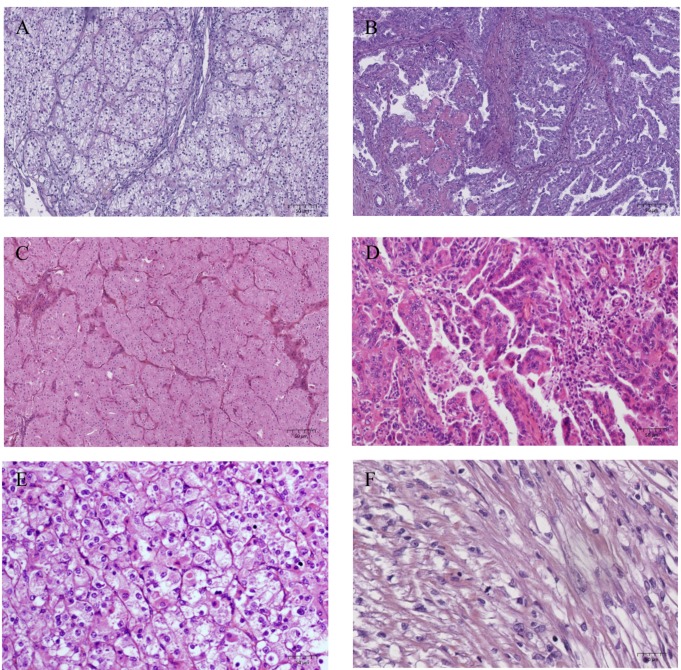
Histological features of renal cell carcinoma, HES staining, x200. (**A**) clear cell renal cell carcinoma, (**B**) papillary renal cell carcinoma, (**C**) chromophobe renal cell carcinoma, (**D**) TFE3 translocation renal cell carcinoma, (**E**) TFEB translocation renal cell carcinoma, (**F**) sarcomatoid component.

Clear cell RCC (ccRCC) is the most frequent histologic entity derived from the proximal convoluted tubule with a morphologic heterogeneity, and is composed of cells with clear or eosinophilic cytoplasm (Figure 1A). These tumours are driven by inactivation of the von Hippel Lindau (*VHL*) tumour suppressor gene [6]. The VHL protein is a multifunctional protein insuring variable regulatory functions, such as remodelling of the extra cellular matrix and control of the cell cycle, but also apoptosis and endocytosis. The function that has been most thoroughly studied is the stability regulation of hypoxia inducible factor (HIF). The VHL protein is a component of an E3 ubiquitin-ligase which targets proteins, including the transcription factor HIF, leading to their ubiquitination and degradation by proteasomes. The consequence of VHL protein impairment is a stabilization of HIF and an increased level of the latter. Such a situation can lead to the transcription of genes regulated by HIF, such as vascular endothelial growth factor (*VEGF*) or carbonic anhydrase IX (*CAIX*) [5]. Consequently, *VHL* acts as one of the main triggers of the angiogenic processes in ccRCC. Other pathways, such as mTOR and MAP kinases pathways, have been described, which also lead to the expression of angiogenic genes [7]. Recently, other common molecular drivers have been identified with mutations commonly involving chromatin-remodelling genes (*PBRM1*, *KDM5C*, *SETD2*, and *BAP1*) [8]. A metabolic shift was also described in aggressive tumours, involving, in particular, the downregulation of genes of the citric acid cycle.

### 2.2. Non-Clear Cell Renal Cell Carcinoma

Papillary renal cell carcinoma (pRCC) is derived from the renal tubular epithelium and represents a heterogeneous disease, including tumours with an indolent outcome (type 1) and tumours with a more aggressive phenotype (type 2) (Figure 1B) [4]. Type 1 papillary renal cell carcinomas are enriched in *MET* alterations, mainly mutations and gains in chromosome 7 involving the *MET* locus. However, *MET* copy number gain is also found in up to 50% of type 2 pRCC. Recurrent alterations of *SETD2*, *EGFR*, *CDKN2A*, *NF2*, *TERT*, and *FH* were also described and occurred more frequently in type 2 pRCC, suggesting deregulation of chromatin remodelling and activation of the cell cycle and MAP kinases pathway [9].

Chromophobe renal cell carcinoma (cRCC) is an eosinophilic tumour (Figure 1C) derived from the distal nephron, characterized by mitochondrial alterations with increased mitochondria count, expression of genes involved the in citric acid cycle, and mutations in mitochondrial DNA copies [10]. They display frequent chromosome loss and less somatic mutations than in other types of RCC. The most frequently mutated gene was *TP53* and the most frequent oncogenic pathway alterations involved mTOR.

Translocation renal cell carcinoma (tRCC) is characterized by gene fusions involving two members of the Microphthalmia-associated transcription factor (MiTF) family: *TFE3* and *TFEB* located on Xp11.2 and 6p21.1 respectively [4,11]. TFE3 tRCC displays a papillary architecture with epithelioid clear cells and psammoma bodies, as shown in Figure 1D,E. TFEB tRCC are biphasic neoplasms with epithelioid cells and smaller cell clusters. TFE3 and TFEB have multiple translocation partners, mainly involved in messenger RNA splicing [12]. In addition, TFE3 tRCC have genome-wide expression alterations that are not found in tumours with *TFEB* gene fusions, suggesting different oncogenic mechanisms between tRCC [13].

Collecting duct carcinoma (CDC) arises from the principal cells of the renal collecting ducts of Bellini and was initially thought to share similarities with upper urinary tract urothelial carcinoma [14,15]. However, molecular analysis revealed that CDCs had distinct copy number alterations, as well as a specific gene expression signature closer to other RCC. They have been identified as immunogenic tumours characterized by the upregulation of genes involved in the immune response, including T-cell activation and proliferation and a high rate of lymphocyte infiltration [5]. Additional reports of CDC genomic alterations highlight the prevalence of the metabolic shift and cell cycle deregulations. Mutations of *NF2*, *SETD2*, and *SMARCB1* were also reported [16].

Renal medullary carcinoma (RMC) is a rare tumour centred on the renal medulla whose current understanding is limited [17]. Several reports highlight a consistent loss of SMARCB1/INI1, a core component of the SWI/SNF chromatin remodelling complex [18].

Sarcomatoid features can occur in each histologic subtype (Figure 1F) and in the sarcomatoid component, demonstrate an increased mutational burden along with a higher frequency of *TP53*, *CDKN2A*, and *NF2* mutations and chromatin remodelling genes, *BAP1* and *ARID1A* [19].

## 3. Anti-Tumour Immune Response and Interaction with Angiogenesis

### 3.1. The Anti-Tumour Immune Response

**Figure 2:**
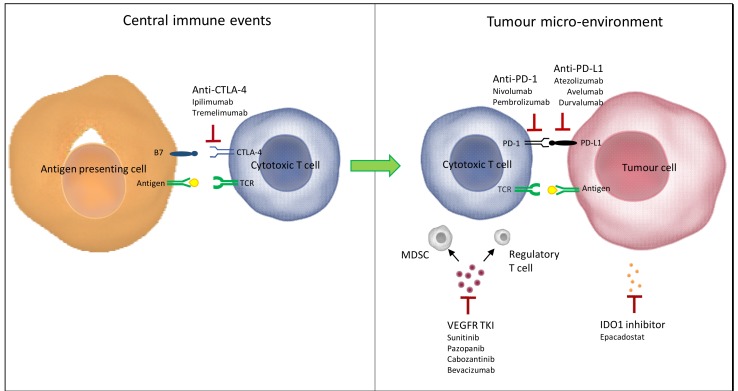
Rationale for combining immune checkpoint inhibitors with antiangiogenic drugs, anti-tumor immune response modulation adapted from Amin et al. *Frontiers in Oncology.* 2019. TCR, T cell receptor; MDSC, myeloid derived suppressor cells; VEGF, vascular endothelial growth factor; PD-1, programmed death 1; PD-L1, programmed death ligand 1; CTLA 4, cytotoxic T lymphocyte antigen 4.

The anti-tumour immune response is based on the cooperation between innate and adaptive immune cells [20]. The first step of the cancer-immunity cycle described by Chen et al. [21] is the release of tumour cell antigens and their capture by dendritic cells (innate immune cells) (Figure 2). Then, in the lymph node, dendritic cells present the captured antigens on MHC I and MHC II to T cells (adaptive immune cells), leading to the activation of effector T cell responses against tumour cells antigens. Through blood vessels, the activated effector T cells migrate to tumours and specifically recognize and bind to tumour cells through the interaction between its T cell receptor (TCR) and its cognate antigen bound to MHC, thus killing antigen-bearing tumour cells.

Unfortunately, in patients, the anti-tumour immune response is not optimal. For instance, tumour antigens may not be detected [22]. Immune cells may consider antigens as self rather than foreign, leading to T regulatory cell responses. In addition, T cells may be inhibited from infiltrating tumour cells [23]. Most importantly, factors in the tumour micro-environment might suppress those effector T cells that are produced.

The activity of T cells is modulated by immune checkpoints expressed on their surface that can generate inhibitory signals. In lymph node sites, the activation of CTLA-4 on T cells when they binds to B7 ligands on antigen-presenting cells decreases T cells and their activity. In tumour sites, PD-1 expressed on activated T cells and upon binding to its ligand PD-L1 on tumour cells, leads to T cell exhaustion. PD-L1 is not only expressed by tumour cells, but also by immune cells, such as macrophages [24].

Biological rationale encouraged the synergy of CTLA-4 inhibition, which favours the development of an active immune response at the level of T-cell proliferation, with PD-1 inhibition, which modulates the immune response at the level of the tumour micro-environment.

### 3.2. Interaction between Angiogenesis and the Anti-Tumour Immune Response

In ccRCC, the inactivation of *VHL* results in an increase of growth factors, such as VEGF, that favour the proliferation and migration of endothelial cells. Increased levels of VEGF in the tumour can induce suppression of both innate and adaptive immune responses. For instance, VEGF have been demonstrated to directly inhibit dendritic cell maturation, decreasing antigen presentation and T cells development, and thus leading to their exhaustion [25,26,27]. In addition, VEGF induce more immune suppression in the tumour micro-environment by increasing the expression of inhibitory checkpoints and by the recruitment of myeloid-derived suppressor cells (MDSC) and T-regulatory cells (Figure 2) [28,29,30]. VEGF also results in neo-angiogenesis with tortuous vasculature and abnormal vessel formation that alters the quality and quantity of infiltration of immune cells in the tumour immune micro-environment.

Interestingly, VEGF inhibitors are able to reverse these effects by improving dendritic cell function and antigen presentation, vasculature normalization with greater trafficking of immune cells, increased cytotoxic T cell infiltration, and decreased MDSC and T-regulatory cells that could potentially reduce the immunosuppressive effect in the tumour micro-environment [31,32].

## 4. Clinical Trials Targeting the PD-L1/PD-1 Pathway

### 4.1. Immune Checkpoint Inhibitors Alone or in Combination

Since the use of interleukin-2 and interferon-alpha in metastatic renal cell carcinoma at the end of the 1990s, the understanding of tumour immunity has significantly improved [33,34]. Nivolumab, a human IgG4 anti-PD-1 antibody, showed its efficacy in metastatic ccRCC in patients previously treated by antiangiogenics, over everolimus (mTOR inhibitor), in a phase III trial, CheckMate 025 (NCT01668784) (Table 2) [35]. The study met its primary endpoint with a significantly longer median overall survival (OS): 25 months with nivolumab versus 19.6 months with everolimus (HR = 0.73; 98.5% CI, 0.57 to 0.93; *p* = 0.0018). The median progression-free survival (PFS) was 4.6 months vs. 4.4 months. Nivolumab also showed benefits in secondary outcomes with a higher objective response rate of 25% versus 5% and a better safety profile compared to everolimus. The expression of PD-L1 was associated with a poorer survival, but was not associated to its response to nivolumab.

Nivolumab was then associated with another immune checkpoint inhibitor: Ipilimumab (anti-CTLA-4) (Table 3) [36,37,38]. In CheckMate 214 (NCT02231749), a phase III study by Motzer et al. confirmed the benefit of the nivolumab and ipilimumab association versus sunitinib in first line metastatic RCC [39]. Patients with intermediate and poor risk according to the International Metastatic Database Consortium (IMDC) score benefitted the most from the association of ipilimumab and nivolumab with a median OS not reached with nivolumab plus ipilimumab versus 26 months with sunitinib (HR = 0.63; 99.8% CI, 0.44 to 0.89; *p* < 0.001). The objective response rate was 42% versus 27%, and the complete response rate was 9% versus 1%. By contrast, the response rate was significantly higher with sunitinib in favourable-risk patients. In an exploratory analysis, a longer PFS was observed in patients with PD-L1 expression. However, the OS and objective response rate (ORR) improvements were demonstrated irrespective of PD-L1 expression. The frequency of treatment-related adverse events was similar in the two arms with less grade 3 or 4 events in the nivolumab plus ipilimumab arm. Nevertheless, treatment-related adverse events leading to discontinuation occurred more frequently in the nivolumab plus ipilimumab group.

A phase 3 trial (NCT03260894) originally proposed the association of epacadostat to pembrolizumab compared to sunitinib. Epacadostat targets and binds to IDO1, an enzyme responsible for the oxidation of tryptophan into kynurenine (Figure 2). By inhibiting IDO1 and decreasing kynurenine in tumour cells, epacadostat restores and increases the proliferation and activation of immune cells, including dendritic cells, natural killer (NK) cells, and T-lymphocytes, as well as interferon production, and a reduction in tumour-associated regulatory T cells.

The effect of immunotherapy could be enhanced by radiotherapy. Tumour destruction by radiotherapy induced the immune response by releasing tumour antigens corresponding to the abscopal effect [40]. The addition of radiotherapy is currently being evaluated in a phase II trial (NCT03469713).

Similar trials in metastatic nccRCC are currently recruiting. In particular, the nivolumab plus ipilimumab association is being evaluated in a first line versus sunitinib in a phase II trial (NCT03075423). Pembrolizumab is also being evaluated in the cohort B of the Keynote 427 phase II trial. In pRCC, durvalumab is being evaluated in combination with savolitinib, a highly selective MET tyrosine kinase inhibitor, in the Calypso phase II trial (NCT02819596). Interestingly, in a retrospective study including metastatic RCC (*n* = 35) with several histologies, nivolumab in monotherapy had an objective response rate of 20%, a median PFS of 3.5 months, and was well tolerated [41]. Out of the four collecting duct carcinomas, one achieved partial response.

It is not surprising that the important results achieved in these trials have encouraged the exploration of immunotherapy in other adjuvant/neo-adjuvant settings to reduce the incidence of progression. Nivolumab was investigated in pre- and post-operative settings in two phase I/II trials and in association with ipilimumab in CheckMate 914 (NCT03138512), a phase III trial, including randomized versus placebo patients with localized renal cell carcinoma who underwent radical or partial nephrectomy with high risk of recurrence. Pembrolizumab and atezolizumab are currently being evaluated in the phase III trials, IMmotion010 (NCT03024996) and Keynote 564 (NCT03142334), respectively.

### 4.2. Association of Immune Checkpoint Inhibitors and Tyrosine Kinase Inhibitors

Combining an immune checkpoint inhibitor and an antiangiogenic drug is a new strategy, with several ongoing trials and published results of phase III trials (Table 3 and Table 4). The difficulty is in finding an association with acceptable toxicity. The Checkmate 016 (NCT01472081), a phase I study, evaluated the safety of the intuitive association of nivolumab plus sunitinib. It resulted in a high frequency of high-grade toxicities, limiting its use in future trials [42].

The phase III study, IMmotion 151 (NCT02420821), explored the after phase II study, IMmotion 150, atezolizumab in association with bevacizumab versus sunitinib [43]. Patients were stratified by PD-L1 status (<1% vs. ≥1% PD-L1 expression on tumour-infiltrating immune cells). The coprimary endpoints were PFS in PD-L1+ patients and OS in intention-to-treat (ITT) patients. The secondary endpoints included PFS in ITT patients, the objective response rate (ORR), and duration of response (DOR). The first results were recently released and even if the OS data are not yet mature (median survival follow-up was 15 months), the PFS in ITT patients with PD-L1+ tumour was 11.2 months versus 8.4 months (HR = 0.74; 98.5% CI, 0.57 to 0.96; *p* = 0.02), which is comparable with PFS in IIT patients irrespective of PD-L1 expression (11.2 months versus 8.4 months), with acceptable safety.

Already used as second-line treatment in metastatic RCC, axitinib, with less hepatotoxicity than sunitinib and pazopanib, could represent a better alternative in association with ICI [44]. The combination of avelumab and axitinib showed encouraging results in phase I and II trials [45]. The phase III trial, JAVELIN Renal 101 (NCT02684006), randomized patients in two arms: Avelumab plus axitinib versus sunitinib [46]. Median PFS was significantly longer in the avelumab plus axitinib group than in the sunitinib group: 13.8 months versus 8.4 months, respectively (HR = 0.69; 95% CI, 0.56 to 0.84; *p* < 0.001). PFS and ORR benefits were seen in all subgroups irrespective of IMDC prognostic risk groups, PD-L1 status, and prior nephrectomy status. However, with a median follow-up of only 12 months, OS data are still pending.

The phase III trial, Keynote 426 (NCT02853331), compared pembrolizumab plus axitinib combination versus sunitinib in first line treatment and showed similar results [47]. The median PFS was longer in the pembrolizumab plus axitinib arm with 15.1 months versus 11.1 months (HR = 0.69; 95% CI, 0.57 to 0.84; *p* < 0.001). The safety profile was comparable to the results of the JAVELIN Renal 101 trial. Interestingly, the benefit of pembrolizumab plus axitinib for OS, PFS, and ORR was observed in the entire population irrespective of the prognostic group and PD-L1 tumour expression.

Similar to the IMmotion 151 trial (NCT02853331), a phase II trial (NCT02724878) is evaluating the combination of atezolizumab plus bevacizumab in metastatic nccRCC. Nivolumab plus cabozantinib was also originally tested in this population (NCT03635892). Taking into account the particularity of TFE/translocation RCC, a phase II trial (NCT03595124) with the combination of nivolumab plus axitinib is currently recruiting.

## 5. Biomarkers and their Limitations

### 5.1. Response Evaluation for Immunotherapy

The version 1.1 of RECIST (RECISTv1.1) is widely used for assessing treatment response [48]. However, these criteria need to be refined regarding the atypical pattern of response to ICI [49]. Indeed, the appearance of new lesions or the growing of established lesions considered by RECISTv1.1 as progressive disease can be observed before an objective response or stable disease. New criteria, all adapted from RECISTv1.1, such as immune-related response criteria (irRC) and, subsequently, irRECIST, immune-based therapeutics RECIST (iRECIST), and immune-modified RECIST (imRECIST), were therefore proposed to prevent misclassification of atypical response as early progression according to RECISTv1.1 [50,51,52,53]

Although it is increasingly accepted that response per immune-related criteria can more accurately assess benefits from immunotherapy, RECISTv1.1 remains the radiological evaluation standard in clinical trials using ICI. The endpoints defined by RECISTv1.1 could potentially impair the identification of predictive biomarkers. For instance, in the CheckMate 010 phase II trial, tumour cell PD-L1 expression was not associated with PFS or ORR defined by RECISTv1.1, but was associated with a longer median irPFS and higher irORR using irRECIST [54]. A high level of T cells with the CD8+ PD-1+ TIM3- LAG3- phenotype, corresponding to T cell activation, was associated with longer median irPFS and higher irORR. Notably, the combination of PD-L1 expression with high levels of T-cell activation identified three groups of patients with significantly different irPFS and irORR.

### 5.2. Immunohistochemical Markers

Predictive biomarker research to select patients eligible for immunotherapy has mainly focused on PD-L1 immunohistochemistry (IHC). Even if PD-L1 was confirmed to be a poor prognostic factor, its use as a predictive marker remains controversial [55]. First, several antibodies are available because a companion antibody corresponds to each drug (Table 1). Studies of the comparability demonstrated variable concordance with interchangeability of the 22C3 and 28-8, but a lower sensitivity of the SP142 assay for evaluating tumour cells [56]. Second, although most trials in RCC considered the percentage of tumour cells, trials evaluating atezolizumab focused on immune cells with different thresholds [43]. Third, PD-L1 is a dynamic biomarker, evolving over time, and its expression is remodelled by the use of antiangiogenic drugs. Fourth, intratumour heterogeneity of PD-L1 and its differential expression between primary RCC and metastases was demonstrated [57,58].

PD-L1 is well known as a ligand localized on the membrane surface. However, there is another isoform of PD-L1; a splice variant generated by splicing out exon 2 encoding an immunoglobulin variable domain (Igv)-like domain was described [59]. However, all other exons are retained without a frameshift. Consequently, the putative translated protein contains all other domains, including the transmembrane region, except for the Igv-like domain. This novel isoform shows a pattern of intracellular membrane that is not able to interact with the PD-1 receptor. Indeed, the anti-PD-L1 antibodies (clone E1L3N, research use only and Dako 28-8) has the ability to detect this non-functional isoform because they target a sequence common to all expressed isoforms within the fourth exon of the *PD-L1* gene [60]. An alternative method of detection of PD-L1, such as flow cytometry, could more accurately characterize the presence of PD-L1 ligand isoforms.

Although most studies used anti-PD-1 drugs, exploratory analyses focused on the ligand PD-L1 expression rather than the receptor PD-1. The expression of PD-1 in tumour-infiltrating lymphocytes was not associated with poor clinical outcomes [61]. If not reported alone as a biomarker in metastatic ccRCC, PD-1 was described in association with other markers to define a T cell activation phenotype correlated with the response to nivolumab [54]. 

In addition, PD-L2 is another ligand competing to bind to PD-1. While its structure is quite similar to PD-L1, the exact function of PD-L2 is unclear, but it could play a role in regulating the T cell immune response and immune tolerance. PD-L2 expression was previously described in RCC and could be evaluated as a predictive biomarker as suggested in other malignancies [57,62].

Interestingly, the expression of PD-L1 was previously correlated to c-MET expression [62,63,64]. This association gives an argument for the combination of c-MET inhibitors and targeted immunotherapy. Indeed, a phase II trial evaluated the safety of the combination of cabozantinib and avelumab and a phase III trial evaluated its association to nivolumab versus sunitinib in first line treatment.

The tumour cells and immune cells of nccRCCs can also express PD-L1. In 101 primary nccRCC, PD-L1expression on tumour cells and immune cells was observed in pRCC (10% and 60%), cRCC (5.6% and 36%), tRCC (30% and 90%), and CDC (20% and 100%) [65]. In concordance with the results on ccRCC, the expression of PD-L1 on tumour cells was associated with worse outcomes in nccRCC. The high expression of PD-L1 in CDC is favoured by the high lymphocytic infiltration and the increased expression of immune response genes in these tumours.

### 5.3. Gene Expression Signatures Related to ICI

Transcriptomic signatures have been developed for patients with first-line TKI (sunitinib or pazopanib) and four different molecular subgroups associated to response and survival have been identified [66,67].

In IMmotion150, exploratory analyses demonstrated that angiogenesis, T-effector/IFN-γ response, and myeloid inflammatory gene expression signatures were associated with clinical outcomes. In particular, atezolizumab monotherapy or therapy combined with the bevacizumab arm was more effective in tumours with the T-effector/IFN-γ response gene expression signature [43].

Interestingly, the BIONIKK randomized phase II trial originally stratified patients according to the transcriptomic signatures of ccRCC (ccrcc1, 2, 3, 4) according to Beuselinck et al. [66]. For instance, the ccrcc4 molecular subtype morphologically showed sarcomatoid differentiation with a strong inflammatory, Th1-orientation, which promotes cytotoxic responses, but a suppressive immune micro-environment with high expression of PD-1 and its ligands. In this molecular subgroup, nivolumab alone or combined with ipilimumab was administered.

### 5.4. Tumour Mutational Burden

One emerging biomarker of the response to ICI is the tumour mutational burden (TMB), defined by the total number of mutations per coding area of the tumour genome [68]. Among mutations, insertions/deletions, a rich source of immunogenic neoantigens, are more frequently observed in RCC. One hypothesis is that RCC with the worst clinical prognosis better responds to ICI due to a higher mutational load. Unfortunately, differently than expected, the mutational load does not seem to correlate with prognostic groups (IMDC and MSKCC) [69].

Regarding clinical trials, the exploratory biomarker analyses of IMmotion150 indicated that TMB was not associated with PFS [43]. A phase II trial, NIVES (NCT03469713), currently recruiting, is evaluating nivolumab plus radiotherapy and will assess the TMB in RCC and its impact on the response to therapy.

Regarding nccRCC, median mutation tumour burdens in the pRCC and ccRCC range are comparable between 1 and 10 per megabase. In contrast, cRCC displays a lower tumour burden between 0.1 and 1 mutation per megabase [70].

## 6. Conclusions

Renal cell carcinoma encompasses distinct diseases and molecular alterations with ccRCC the most frequent entity characterized by its angiogenesis and its immune micro-environment targeted by antiangiogenic drugs and ICI.

In metastatic ccRCC, unprecedented OS, PFS, and response rates were reached using a combination of ICI (anti-CTLA-4 and anti-PD1) for intermediate and poor risk patients. The PFS was even improved by the combination of ICI with antiangiogenic drugs, even for favourable risk patients, with preliminary results of pivotal phase III clinical trials recently released. However, the best complete response rate was reached with ICI combination. Future trials will have to change the control arm to ipilimumab plus nivolumab or ICI plus antiangiogenic drugs instead of sunitinib. In adjuvant settings, ICI are also promising, with ongoing clinical trials.

Even if clinical trials evaluating ICI are also developed in nccRCC, the specificity of each entity is rarely taken into account. Indeed, because the entities are rare, they usually include different entities in the same trial. As proposed for tRCC, we emphasize the need of subtype specific clinical trials, taking into account the distinct molecular alterations in each entity, to allow a personalized approach.

The selection of patients who will respond specifically to ICI remains a challenge. Data from the clinical trials in RCC show that the use of PD-L1 expression alone is insufficient to predict treatment response. The clinical utility of mutational burden has not yet been demonstrated in RCC studies and is still under evaluation and the proportion of insertions and deletions could be more relevant in RCC [71]. The gene expression signatures are promising and currently developed for patients who benefited from ICI.

The future of predictive biomarkers will probably involve a combination of different biologic variables as it is clear that a single biomarker may not be enough to determine treatment response, especially in the context of combined drugs. These composite biomarkers will need to be prospectively validated in the context of therapeutic clinical trials.

## Figures and Tables

**Table 1:** Checkpoint inhibitors and corresponding IHC antibodies used in clinical trials for renal cell carcinoma.

Checkpoint Inhibitor	Target	Antibody for PD-L1 IHC	PD-L1 Assessment
Ipilimumab	CTLA-4	Not applicable	Not applicable
Nivolumab	PD-1	Dako 28-8	% tumour cells
Pembrolizumab	PD-1	Dako 22C3	% tumour cells
Atezolizumab	PD-L1	Ventana SP142	% immune cells
Durvalumab	PD-L1	Ventana SP263	Not available
Avelumab	PD-L1	Dako 7310	% immune cells

Abbreviations: CTLA-4, cytotoxic T-lymphocyte associated protein 4 antibody; PD-1, programmed cell death protein 1; PD-L1, programmed death-ligand 1

**Table 2:** Clinical trials evaluating immune checkpoint inhibitors alone or in combination.

NCT Number/Study Name	Targeting Agents	Comparison	Phase	Histology	Primary Endpoint	Therapy Setting	Status
NCT01668784/CheckMate 025	nivolumab	everolimus	III	ccRCC	OS	at least second line	published
NCT02231749/CheckMate 214	Nivolumab + ipilimumab	sunitinib	III	ccRCC	PFS, OS, ORR	first line	published
NCT03260894	Pembrolizumab + epacadostat	sunitinib/pazopanib	III	ccRCC	ORR	first line	active not recruiting
NCT02853344/Keynote-427	pembrolizumab	-	II	cc/nccRCC	ORR	all lines	active not recruiting
NCT02964078	pembrolizumab+interleukin-2	-	II	ccRCC	ORR	all lines	active not recruiting
NCT02960906/BIONIKK	nivolumab/ipilimumab/VEGFR-TKI	-	II	ccRCC	ORR	first line	recruiting
NCT03469713/NIVES	Nivolumab + SBRT	-	II	ccRCC	ORR	at least second line	recruiting
NCT02446860/ADAPTeR	nivolumab	-	II	ccRCC	Safety	pre and post-operative	recruiting
NCT02819596/Calypso	Durvalumab +/tremelimumab/savolitinib	-	II	cc/pRCC	DLT, OR	at least second line	recruiting
NCT03308396	Durvalumab + guadecitabine	-	I/II	ccRCC	safe dose/ORR	at least second line	recruiting
NCT02989714	Nivolumab + interleukin-2	-	I/II	ccRCC	safety	third line	recruiting
NCT03024996/IMmotion010	atezolizumab	placebo	III	ccRCC	DFS	adjuvant	recruiting
NCT03055013	nivolumab	observation	III	ccRCC	DFS	adjuvant	recruiting
NCT03138512/CheckMate 914	Nivolumab + ipilimumab	placebo	III	ccRCC	DFS	adjuvant	recruiting
NCT03142334/Keynote-564	pembrolizumab	placebo	III	ccRCC	DFS	adjuvant	recruiting
NCT02575222	nivolumab	-	I	ccRCC	safety	neo-adjuvant	active not recruiting
NCT03177239/ANZUP1602	Nivolumab + ipilimumab	-	II	nccRCC	ORR	all lines	recruiting
NCT03075423/SUNIFORECAST	Nivolumab + ipilimumab	sunitinib	II	nccRCC	OS	first line	recruiting

Abbreviations: ccRCC, clear cell renal cell carcinoma; nccRCC, non clear cell renal cell carcinoma; OS, overall survival; OR, overall response; PFS, progression-free survival; ORR, objective response rate; DLT, Dose-Limiting Toxicities; -, absence of comparison group.

**Table 3:** Comparison of pivotal phase III clinical trials with available results evaluating immune checkpoints inhibitors.

Study Name	Comparator Arms	N ITT	Median FU	OS ITT	HR (95% CI)	PFS ITT	HR (95% CI)	ORR (%)	CR (%)	Grade ≥ 3 (%)
CheckMate 214	Ipilumab + nivolumab	550	25.2	Nr *	0.63	11.6 *	0.82	42 *	9 *	46
	sunitinib	546		26 *	(0.44–0.89)	8.4 *	(0.64–1.05)	27 *	1 *	35
JAVELIN Renal 101	Avelumab + axitinib	442	11.6	na		13.8	0.69	51.4	3.4	71.2
	sunitinib	446	10.7	na		8.4	(0.56–0.84)	25.7	1.8	71.5
Keynote 426	Pembrolizumab + axitinib	432	12.8	na		15.1	0.69	59.3	5.8	75.8
	sunitinib	429		na		11.1	(0.57–0.84)	35.7	1.9	70.6
IMmotion 151	Atezolizumab + bevacizumab	454	15	na		11.2	0.83	37	5	40
	sunitinib	461		na		8.4	(0.70–0.97)	33	2	54

Abbreviations: ITT, intention-to-treat; OS, overall survival; FU, follow-up; HR, Hazard Ratio; PFS, progression-free survival; ORR, objective response rate; CR, complete response; nr, not reached; * in intermediate and poor-risk patients according to IMDC score; nr, not reached; na, not available.

**Table 4:** Clinical trials evaluating immune checkpoint inhibitors in association with antiangiogenic drugs .

NCT Number/Study Name	Targeting Agents	Comparison	Phase	Histology	Primary Endpoint	Therapy Setting	Status
NCT02684006/Javelin Renal 101	Avelumab + axitinib	sunitinib	III	ccRCC	PFS, OS	first line	published
NCT02853331/Keynote 426	Pembrolizumab + axitinib	sunitinib	III	ccRCC	PFS, OS	first line	published
NCT02420821/IMmotion 151	Atezolizumab + bevacizumab	sunitinib	III	ccRCC	PFS, OS	first line	active not recruiting
NCT02811861/CLEAR	Lenvatinib + everolimus/pembrolizumab	sunitinib	III	ccRCC	PFS	first line	recruiting
NCT03141177/CheckMate 9ER	Nivolumab + cabozantinib	sunitinib	III	ccRCC	PFS	first line	recruiting
NCT01984242/IMmotion 150	Atezolizumab +/- bevacizumab	sunitinib	II	ccRCC	PFS	first line	Published
NCT02014636/Keynote-018	Pembrolizumab + pazopanib	pazopanib/pembrolizumab	II	ccRCC	safety, efficacy	first line	active not recruiting
NCT02348008	Pembrolizumab + bevacizumab	-	I/II	ccRCC	safe dose, efficacy	at least second line	active not recruiting
NCT02348008	Pembrolizumab + bevacizumab	-	I/II	ccRCC	safe dose, efficacy	at least second line	active not recruiting
NCT03024437	Atezolizumab + bevacizumab + entinostat	-	I/II	ccRCC	safe dose, ORR	at least second line	recruiting
NCT03172754	nivolumab + axitinib	-	I/II	ccRCC	safety	at least second line	recruiting
NCT02493751/Javelin Renal 100	Avelumab + axitinib	-	I/II	ccRCC	DLT	first line	Published
NCT02501096	Pembrolizumab + lenvatinib	-	I/II	ccRCC	DLT, ORR	all lines	recruiting
NCT03200587	Avelumab + cabozantinib	-	Ib	ccRCC	safe dose	all lines	recruiting
NCT01472081/CheckMate 016	Nivolumab + sunitinib/pazopanib/ipilimumab	nivolumab	I	ccRCC	safety, tolerability	at least second line	Published
NCT02133742	Pembrolizumab + axitinib	-	I	ccRCC	DLT	first line	Published
NCT02724878	Atezolizumab + bevacizumab	-	II	nccRCC	ORR	all lines	recruiting
NCT03635892	Nivolumab + cabozantinib		II	nccRCC	ORR	all lines	recruiting
NCT03595124	Nivolumab + axitinib	-	II	tRCC	PFS	all lines	recruiting

Abbreviations: ccRCC = clear cell renal cell carcinoma, nccRCC = non clear cell renal cell carcinoma, pRCC = papillary renal cell carcinoma, tRCC = translocation renal cell carcinoma, OS = overall survival, PFS = progression-free survival, ORR = objective response rate, DLT = Dose-Limiting Toxicities, - absence of comparison group.
